# Cotinine and Polycyclic Aromatic Hydrocarbons Levels in the Amniotic Fluid and Fetal Cord at Birth and in the Urine from Pregnant Smokers

**DOI:** 10.1371/journal.pone.0116293

**Published:** 2014-12-30

**Authors:** Julia de Barros Machado, José Miguel Chatkin, Aline Rigon Zimmer, Ana Paula Szezepaniak Goulart, Flávia Valladão Thiesen

**Affiliations:** 1 School of Medicine, Pontifícia Universidade Católica, Rio Grande do Sul, Porto Alegre, Brazil; 2 School of Pharmacy, Pontifícia Universidade Católica, Rio Grande do Sul, Porto Alegre, Brazil; University of Rochester Medical Center, United States of America

## Abstract

Cigarette smoking during pregnancy has several impacts on fetal development, including teratogenic effects. The objective of this study was to assess whether the toxic substances (cotinine and polycyclic aromatic hydrocarbons) found in pregnant smokers are transmitted to their fetuses. The outcomes were analyzed measuring cotinine and 1-hydroxypyrene in the amniotic fluid and maternal urine, benzopyrene and cotinine in the umbilical cord blood. Through a controlled cross-sectional design, 125 pregnant women were selected and classified according to their smoking status: 37 current smokers, 25 passive smokers and 63 non-smokers (controls). We performed high-performance liquid chromatography to measure substances’ concentrations. A post-hoc Tukey’s test was used to analyze the differences between the groups. All variables were significantly different between controls and smokers. The mean ratios between the concentration of cotinine in smokers compared to controls were as follows: 5.9 [2.5–13.5], p<0.001 in the urine; 25 [11.9–52.9], p<0.001 in the amniotic fluid; and 2.6 [1.0–6.8], p = 0.044 in the umbilical cord blood. The mean ratios of 1-hydroxypyrene concentration between smokers and controls were 7.3 [1.6–29.6], p = 0.003 in the urine and 1.3 [1.0–1.7], p = 0.012 in the amniotic fluid, and of benzopyrene in umbilical cord blood was 2.9 [1.7–4.7], p<0.001. There were no significant differences between controls and passive smokers. When comparing the three groups together, there were statistical differences between all variables. Thus, the fetuses of pregnant smokers are exposed to toxic and carcinogens substances. To our knowledge, this is the first study to measure 1-hydroxypyrene in the amniotic fluid and benzopyrene in umbilical cord blood by high-performance liquid chromatography when considering pregnant women in relation to smoking exposure only.

## Introduction

Smoking is the leading cause of preventable deaths in the world [1], [2], [3], causing the deaths of approximately 6 million people per year [4], [5]. Cigarette smoking before and during pregnancy is an important cause of preventable illness and death among mothers and their children and particularly impacts on pregnancies in younger women with lower educational level [6]. Smoke exposure during pregnancy has been repeatedly and consistently related to numerous risks to the fetus, including low birth weight, preterm delivery, increased risk of miscarriage [7], [8], [9], [10], [11], [12], [13], and increased teratogenic effects on the development of several organs [14].

The ultrasound pattern of maternal-fetal chronic hypoxia has already been described in pregnant smokers [15]. Placental abruption, which occurs in approximately 1% of pregnancies, is 20 to 30 times more frequent in pregnant smokers [16]. Also, cigarette smoke contains numerous carcinogens that cross the placenta [17], increasing the risk of childhood cancer, such as brain tumors, leukemia and lymphoma [18].

Cotinine, nicotine metabolite [19], [20], [21], is widely used as a biological marker to measure tobacco exposure [22], [23], [24] and is also used in cases of low or intermittent tobacco use [25]. It is also considered the best marker of smoking status, even during pregnancy [26].

Polycyclic aromatic hydrocarbons (PAHs) are related to the teratogenic and carcinogenic effects of cigarette smoke. PAHs are a class of organic compounds containing two or more fused aromatic rings consisting of carbon atoms and hydrogen [27]. These substances are air pollutants generated by burning tobacco leaves, motor vehicles, and factory combustion and can also be found in some smoked foods [28]. Most PAH compounds have an 18-hour half-life [29], [30] and are metabolized and excreted in the urine. Benzopyrene and 1-hydroxypyrene can be used as biomarkers of recent tobacco exposure [31].

Llop et al. [32] demonstrated that one of the PAHs, urinary 1-hydroxypyrene, is a good marker for assessing exposure to cigarette smoke and air pollution. When the determination is performed on the blood, the biomarker used is benzopyrene. After inhalation, the PAHs are metabolized to form reactive epoxide and phenolic compounds that have the capacity to bind to DNA, forming complex PAH-DNA adducts [33]. These PAH-DNA adducts increase the probability of genetic mutations and, therefore, might be associated with several forms of cancer [34].

Smokers excrete significantly higher urine levels of 1-hydroxypyrene than nonsmokers, and these values correlate well with the number of cigarettes smoked [35]. However, only a few studies have investigated the levels of PAHs in pregnant smokers [36], and the majority of these studies have focused on environmental exposure [32], [35]. Considering that cigarette smoke is the primary source of exposure to these substances, this area of study requires further investigation.

The metabolism of pregnant smoker is altered, and it is difficult to evaluate the effects of dietary factors and environmental exposure. Thus, it is necessary to compare women from the same population with similar diets and exposures to environmental pollution to try to isolate the true exposure of fetuses to chemicals arising from both active and passive smoking.

The objective of this study was to assess whether cotinine and PAH found in pregnant smokers are transmitted to their fetuses. We measured the amount of 1-hydroxypyrene and cotinine in the amniotic fluid and maternal urine and benzopyrene and cotinine in the umbilical cord blood.

## Materials and Methods

In a controlled, cross-sectional design study, we recruited pregnant women in labor admitted at the Obstetric Center Hospital São Lucas (HSL), Pontificia Universidade Catolica do Rio Grande do Sul, Porto Alegre, Brazil, from July 2010 to July 2013. This project was submitted and approved by the PUCRS Research Ethics Committee under the number 10/05066.

The inclusion criteria were signing the informed consent; age 18 to 35 years old; women must had received regular prenatal care at the Department of Obstetrics at the same hospital; and no previous or concomitant gestational diseases. Smokers were defined as pregnant women who had smoked more than 100 cigarettes throughout life and were smoking at the time of the interview [37]. Passive smokers were defined as non-smoking pregnant women who lived with a smoker who smoked inside their home. Non-smokers served as the control groups. The exclusion criteria were patients who declined to participate in the study; women with severe psychopathic disorders; preterm labor; illiteracy; and addiction to other licit or illicit drugs. Former smokers were also excluded.

The volunteers in labor were recruited at the moment of admission to the HSL Obstetric Center. They completed a questionnaire regarding their demographics and smoking habits. Maternal urine was then collected. Upon rupture of the membranes, amniotic fluid was collected. During delivery, the umbilical cord blood sample was collected.

The cotinine in maternal urine and umbilical cord blood were analyzed according to the methods described by Cattaneo *et al* [38] and Petersen *et al*. [39] respectively. The quantitative methodologies for the determination of cotinine and 1-hydroxypyrene in amniotic fluid, as well as, the 1-hydroxypyrene (1-OHP) in the maternal urine and benzopyrene in the umbilical cord blood were previously validated by our research group in accordance with the International Conference on the Harmonization (ICH) guideline [40]. To assess the specificity of the methodologies blank samples (or drug-free human samples) of urine, amniotic fluid and umbilical cord blood were compared to spiked samples with cotinine, 1-hydroxypyrene or benzopyrene and no interference of the different biological matrices was seen in the peak determination. The calibration curves for each analyte were linear (r>0.99) over the concentration ranges of 10.0–1000 µg/L for cotinine in urine; 5.0–500 µg/L for cotinine in umbilical cord blood; 2.0–600 µg/L for cotinine in amniotic fluid; 0.1–10.0 µg/L for 1-OHP in urine; 0.25–15.0 µg/L for 1-OHP in amniotic fluid; and 0.1–7.5 µg/L for benzopyrene in umbilical cord blood. The limits of quantification (LOQ) were defined as the lowest point of calibration curves, and limits of detection (LOD) were calculated as the minimum concentration providing chromatographic signals 3 times higher than the background noise. The analytical methods fulfilled the acceptance criteria for precision and accuracy showing a coefficient of variation lower than 13.4% and a recovery range of 91.5–110%.

The specimens for the cotinine analysis were alkalinized with 25 µL of sodium hydroxide 10 M and submitted to liquid-liquid extraction with dichloromethane using 2-phenylimidazole as internal standard (IS). For each analysis 2 mL of maternal urine, 600 µL of umbilical cord blood, and 1 mL of amniotic fluid were employed.

To determine 1-hydroxypyrene, 2.5 mL of urine samples were treated with acetate buffer (5 mL, pH 5) and β-glucuronidase/arylsulfatase (10 µL), to promote the hydrolysis of urinary 1-hydroxypyrene, and subsequently submitted to solid phase extraction (SPE) using a C18 cartridge. For the analysis of 1-hydroxypyrene in the amniotic fluid 2 mL of sample was submitted to liquid-liquid extraction with ethyl ether. To the determination of benzopyrene in the umbilical cord blood 150 µL of sample was extracted with cyclohexane. The organic phase was separated and dried under nitrogen flow at room temperature. Then, the samples were recovered with mobile phase and injected into the HPLC system.

Cotinine analysis was performed using an high-performance liquid chromatograph equipped with a UV detector isocratic pump, manual injection and software. The chromatographic separation was performed (4.6×150 mm×5 µm), column protected (4.6×12.5 mm, 5 µM). The mobile phase consisted of a mixture of water:methanol:sodium acetate (0.1 M):acetonitrile (50∶15∶25∶10, v/v/v/v), and the flow was maintained at 0.5 mL/minute with UV detection at 260 nm, yielding a total run time of 10 minutes.

A high-efficiency liquid chromatograph equipped with a fluorescence detector, isocratic pump and manual injector was used to analyze the presence of 1-hydroxypyrene and benzopyrene. The chromatographic separation was performed with (4.6×150 mm, 5 µM) column protected by a guard column (4.6×12.5 mm, 5 µM). The mobile phase consisted of a methanol:acetonitrile:water (35∶35∶30, v/v/v) mixture for 1-hydroxypyrene, and acetonitrile:water (75∶25, v/v) for benzopyrene analysis. A 1.0 mL/min flow rate was maintained isocratically with fluorescence detector set at 242 nm (excitation) and 388 nm (emission) for 1-hydroxypyrene, and 290 nm (excitation) and 430 nm (emission) for benzopyrene (see also S1 Table).

To characterize the patients and fetal birth we used means and standard deviations. For analyses the group comparisons of cotinine and PAHs concentration according to smoking habit, the quantitative variables were logarithmically transformed and presented as geometric means and geometric mean deviation. The difference between the 3 groups together was calculated using ANOVA.

To estimate the proportional difference of asymmetrical variables between groups, we used the mean ratio (MR) and 95% confidence interval (CI). In relative terms, the MR expresses how many times the average of a group is larger than the other. The MR was obtained in a model of analysis of covariance, and a robust standard error was applied to the logarithms of the measures. The post-hoc Tukey’s test was used to determine the group differences, letters a & b denote non-significant or significant difference between groups, respectively. The groups with no significant difference were labeled as (a) and the groups, with significant difference as (b). The significance level was 0.05. The data were analyzed using SPSS version 21.0.

## Results

A total of 125 patients were enrolled in the study: 37 (29.6%) were smokers; 25 (20%) were passive smokers; and 63 (50.4%) were non-smokers. There were no significant differences in age or obstetric aspects between the groups (Table 1).

**Table 1:** Patients and fetal birth characteristics.

Characteristics	Control(n = 63)	Passive Smokers(n = 25)	Smokers(n = 37)	P
**Age (years)**	26+/−5	24+/−6	26+/−5	0.24
**BMI (kg/m^2^)**	27+/−5	28+/−5	28+/−4	0.49
**Pregnancies**	2+/−1	2+/−2	2+/−1	0.17
**Gestational age (weeks)**	38+/−1	38+/−1	39+/−1	0.15
**Weight (g)**	3349+/−409	3375+/−433	3225+/−528	0.33
**Apgar (5-minute)**	9+/−0.7	9+/−0.6	9+/−1.0	0.09

Data are presented as means +/− standard deviations. BMI (Body Mass Index). Apgar score index. is a method for assessing a neonate’s heart rate, respiratory effort, muscle tone, skin color, and reflex irritability.

Table 2 presents the cotinine and 1-hydroxypyrene levels in the maternal urine and amniotic fluid and the benzopyrene and cotinine levels in the umbilical cord blood.

**Table 2:** Group comparisons of selected variables according to smoking habit.

	Controls	PassiveSmokers	Smokers	MR(PS/C)	MR(S/C)	P
Urinary Cotinine (µg/L)	n = 56	n = 25	n = 35	1.4[0.5–3.7]	5.9[2.5–13.5]	<0.001
	0.64^a^(ND-68)	0.94^a^(ND-21)	3.77^b^(ND-69)			
Amniotic Fluid (µg/L)	n = 55	n = 22	n = 33	0.8[0.3–2.0]	25[11.9–52.9]	<0.001
	1.47^a^(ND-63)	1.28^a^(ND-51)	36.87^b^(ND-527)			
Umbilical CordCotinine (µg/L)	n = 59	n = 24	n = 37	0.9[0.32–2.9]	2.6[1.0–6.8]	0.038
	3.36^a^(ND-91)	3.23^a,b^(ND-84)	8.79^b^(ND-340)			
1-hidroxypyrene inmaternal urine(µmol/mol creatinine)	n = 60	n = 25	n = 37	2.4[0.4–11.9]	7.3[1.6–29.6]	0.004
	0.02^a^(ND-2.2)	0.05^a^(ND-1.2)	0.15^b^(ND-3)			
1-hidroxypyrene inamniotic fluid (µg/L)	n = 55	n = 22	n = 33	1.0[0.8–1.3]	1.3[1.0–1.7]	0.015
	0.48^a^(ND-1.1)	0.51^a,b^(ND-1.1)	0.67^b^(0,3–1.4)			
Benzopyrene inumbilical cord (µg/L)	n = 51	n = 20	n = 26	1.3[0.7–2.2]	2.9[1.7–4.7]	<0.001
	0.39^a^(ND-1.8)	0.51^a^(0.1–1.8)	1.13^b^(0.1–4.3)			

The data are presented as geometric means (minimum-maximum) or mean ratios (MRs) and [95% CI]. P: statistical significance obtained from analysis of variance model with robust standard error applied to the logarithms of the measurement. Legend: C, control; PS, passive smoker; S, smoker; MR mean ratio, ND not detected. Letters denote significant difference between groups (Tukeýs post-hoc test). a: without statistical significant difference (similar groups), b: groups with statistical difference.

When compared the three groups together were statistical difference between all variables (P in table 2). When compared in pair all variables also were significantly different between the control and smoker groups, represented by letter b in Table 2. There were no significant differences between controls and passive smokers, represented by letter a in Table 2: urinary cotinine P = 0.57, amniotic fluid cotinine P = 0.92, umbilical cord cotinine P = 0.99, 1-hydroxypyrene in maternal urine P = 0.39, 1-hydroxypyrene in amniotic fluid P = 0.86 and benzopyrene in umbilical cord P = 0.47.

The number is not the same in all groups because some samples not able to be analyzed.

The MRs of the cotinine concentrations of pregnant smokers compared to controls were 5.9 [95%CI 2.5 to 13.5] (p<0.001) in the urine; 25 [95%CI 11.9–52.9] (p<0.001) in the amniotic fluid; and 2.6 [95%CI 1.0–6.8] (p = 0.044) in the umbilical cord blood.

The MRs of the 1-hydroxypyrene concentrations in pregnant smokers compared to controls were 7.3 [95%CI 1.6–29.6] (p = 0.003) in the urine and 1.3 [95%CI 1.0–1.7] (p = 0.012) in the amniotic fluid. The concentration of benzopyrene in the umbilical cord blood of fetuses from mothers who smoked had an MR of 2.9 [95%CI 1.7–4.7] (p<0.001) compared to controls.

In Table 3 data of groups are presented as geometric mean (GM) and geometric standard deviation (GSD).

**Table 3:** Geometric mean and standard geometric deviation of groups.

	Controls (C)		PassiveSmokers (PS)		Smokers (S)	
	GM	GSD	GM	GSD	GM	GSD
Urinary Cotinine(µg/L)	0.64	4.33	0.94	4.31	3.77	6.90
Amniotic Fluid Cotinine(µg/L)	1.47	2.56	1.28	2.28	36.87	9.07
Umbilical Cord Cotinine(µg/L)	3.36	6.34	3.23	6.20	8.79	7.83
1-hydroxypyrene inmaternal urine(µmol/mol creatinine)	0.02	17.04	0.05	9.44	0.15	9.63
1-hydroxypyrene inamniotic fluid (µg/L)	0.48	1.65	0.51	1.65	0.67	1.39
Benzopyrene inumbilical cord (µg/L)	0.39	2.71	0.51	2.08	1.13	1.99

Data are presented as geometric mean (GM) and geometric standard deviation (GSD).

## Discussion

Cotinine and polycyclic aromatic hydrocarbons in the urine and amniotic fluid of pregnant smokers and umbilical cord blood of their fetuses had significant higher levels compared to the non-smokers (controls). There were no significant differences between passive smokers and controls. When comparing the three groups together were statistical differences between all variables.

To our knowledge, this study is the first to measure 1-hydroxypyrene in the amniotic fluid and benzopyrene in umbilical cord blood by high-performance liquid chromatography when considering pregnant women in relation to smoking exposure only.

Our results demonstrate that pregnant smokers expose their fetuses to several substances in cigarette smoke, such as nicotine/cotinine and PAHs.

The cotinine concentrations in pregnant smokers were approximately 6 times higher in the urine, 25 times higher in the amniotic fluid, and 2.6 times higher in the cord blood when compared to non-smokers (control group).

The concentrations of 1-hydroxypyrene in pregnant smokers were seven times higher in the urine and 30% higher in the amniotic fluid compared to the control group. The benzopyrene concentration was approximately 3 times higher in cord blood of fetuses of the smokers compared to the control group.

Thus, these substances found in the urine of pregnant smokers pass to the fetus through the amniotic fluid and umbilical cord blood. The process of amniotic fluid formation, in which the fetus urinates and swallows his own urine, forms a cycle in which they are more exposed to these substances. The higher concentration of cotinine in the amniotic fluid compared to the concentration of 1-hydroxypyrene can be explained by its longer half-life (36 hours versus 18 hours, respectively) [29], [41]. The non-significant difference among passive pregnant smokers and controls is probably related that they do not inhale enough cigarette smoke to transmit to the fetus such toxic substances.

We classified the patient smoking status according to their self–report because many smokers were abstinent for many hours before labor. Thus, the smoking status could be affected if it were based on the cotinine levels only (due to the half-life rating). Also, many participants did not smoke when the pain of labor contractions began. Some of the collected samples could not be analyzed by having insufficient amount of material for analysis.

For the statistical analyses, we calculated the mean and median, but the results were not satisfactory due to the intense asymmetry data. We then used the geometric mean and geometric mean ratios, more appropriate tools for studying asymmetric data and values close to zero. We also calculated the geometric standard deviation, but to interpret the values is necessary perform logarithmic transformation and then Gauss (mean +/−1.96 SD).

Our results confirms that nicotine cross the placenta and is already known that it can cause extensive damage to the fetus, since nicotine is clearly neuroteratogenic and impacts the fetal brain at critical stages of development in pregnant women using tobacco [42]. This may explain the cognitive, emotional and behavioral problems observed in these children. Furthermore, exposure to cigarette smoke throughout prenatal and postnatal development increases the likelihood of dependence on licit and illicit drugs in adolescence or adult age [43]. The development of other organs, including the lungs, is also adversely affected by nicotine [44], [45].

We also found an increased fetal exposure to 1-hydroxypyrene, a known carcinogenic substance. The carcinogenic pathway for PAHs or their metabolites involves the production of reactive oxygen species, which generate oxidative stress, lipid peroxidation, protein modifications, and DNA damage, and may influence birth outcomes and child health in later life [46], [47], [48], [49].

Perera et al. [50] showed that a range of relevant environmental exposures to certain carcinogenic PAHs during pregnancy may damage the fetal DNA through histone modification, which may result in fetal chromosomal abnormalities. PAH-DNA adducts were detected in the umbilical cord blood and maternal blood after exposure to ambient air PAHs [50] and predisposes the fetus to aberrations in the cord blood. Thus, prenatal exposure to PAHs may increase the risk of cancer in humans [51].

The early embryonic period, when the rates of DNA synthesis are high and the patterns of DNA methylation are established [52], may be a particularly sensitive phase for epigenetic dysregulation due to environmental exposures.

Perera et al. [50] proposed that the transplacental transfer of PAHs to the fetus could have significant impacts on fetal development. Several studies found a reduction in head circumference at birth, which is correlated with a lower intelligence quotient and poor cognitive functioning and school performance in childhood [53], [54]. Furthermore, PAHs are associated with restricted intrauterine growth [55], small gestational age [56] and preterm delivery [56]. When a group of newborns monitored prenatally was followed through school age, there was a decline in neurological development [47] and an increased likelihood of asthma-related symptoms [57].

Jules et al. [58] suggests that intrauterine exposure to benzopyrene predisposes newborn rats to functional deficits in cardiovascular development, which may contribute to cardiac dysfunction throughout life. Rundle et al. [59] suggests that prenatal exposure to PAHs causes increased fat mass gains during childhood and an increased risk of obesity. Langlois et al. [60] showed an association between maternal occupational exposure to PAHs and increased risk of cleft lip with or without cleft palate.

Stejskalova and Pavek [61] confirmed that PAH-DNA adducts in the placenta also led to pregnancy complications, such as preterm labor, intrauterine growth restriction, structural abnormalities, fetal death, placental abruption, low birth weight, and small birth length.

The measurement of PAH and its fetal effects have been extensively studied, as mentioned above. However, most of these studies focused on environmental exposures.

What this study adds is that we studied the fetal exposure to PAHs solely related to smoking, which is the primary means of exposure, in a population with a similar diet, living in the same geographic area, and without significant variations in pollution exposure. We measured benzopyrene levels in the umbilical cord blood by high-performance liquid chromatography, a simple and inexpensive method. Our results confirmed that benzopyrene also passes to the fetus from cigarette smoke exposure. We also showed that PAHS were also transmitted to the fetus through the amniotic fluid.

Thus, the fetuses of pregnant women who smoke are exposed to notoriously toxic and carcinogenic substances.

## Supporting Information

S1 Table **Parameters of analytical performance.**
(DOCX)
