# Supplementary material for: Cotinine and Polycyclic Aromatic Hydrocarbons Levels in the Amniotic Fluid and Fetal Cord at Birth and in the Urine from Pregnant Smokers
Source: PLoS One. 2014 Dec 30;9(12):e116293. doi: 10.1371/journal.pone.0116293 (PMC4280223; doi:10.1371/journal.pone.0116293)
Supplement: S1 Table — Parameters of analytical performance. (DOCX) [file pone.0116293.s001.docx]

**Table S1 -** Parameters of analytical performance

| **Biomarker/**  **Matrix** | **Range of linearity**  **(ng/mL)** | **Coefficient of correlation (*r*)** | **LOQ**  **(ng/mL)** | **LOD**  **(ng/mL)** | **Precision**  **(CV%)** | **Accuracy**  **(%)** |
| --- | --- | --- | --- | --- | --- | --- |
| Cotinine/  urine | 10.0 –1000.0 | 0.9989 | 10.0 | 5.0 | 8.1-12.3 | 97.0-107.0 |
| Cotinine/  cord blood | 5.0 – 500.0 | 0.9980 | 5.0 | 0.18 | 1.3-11.6 | 97.0-110.0 |
| Cotinine/  amniotic fluid | 2.0 - 600.0 | 0.9992 | 2.0 | 0.50 | 3.9-13.4 | 91.5-101.7 |
| 1-hydroxypyrene/  urine | 0.10 - 10.0 | 0.99989 | 0.10 | 0.045 | 3.2-12.5 | 94.0-102.5 |
| 1-hydroxypyrene/ amniotic fluid | 0.25 - 15.0 | 0.9977 | 0.25 | 0.06 | 8.7-10.4 | 98.0-102.2 |
| Benzopyrene/  cord blood | 0.10 - 7.5 | 0.9965 | 0.10 | 0.04 | 5.1-8.6 | 100.4-108.3 |

LOQ: limit of quantification; LOD: limit of detection; CV: coefficient of variation
